# Influence of Myrrh Extracts on the Properties of PLA Films and Melt-Spun Multifilament Yarns

**DOI:** 10.3390/ma13173824

**Published:** 2020-08-29

**Authors:** Evaldas Bolskis, Erika Adomavičiūtė, Egidijus Griškonis, Valdas Norvydas

**Affiliations:** 1 Faculty of Mechanical Engineering and Design, Kaunas University of Technology, Studentu Str. 56, 51424 Kaunas, Lithuania; erika.adomaviciute@ktu.lt (E.A.); valdas.norvydas@ktu.lt (V.N.); 2 Faculty of Chemical Technology, Kaunas University of Technology, Radvilenu pl. 19, 50254 Kaunas, Lithuania; egidijus.griskonis@ktu.lt

**Keywords:** myrrh, PLA, melt spinning, multifilament yarns

## Abstract

A possible approach for providing new properties for textiles is the insertion of natural ingredients into the textile product during the process of its manufacture. Myrrh has long been used in medicine as an antibacterial and antifungal material. Polylactide (PLA) is a thermoplastic synthetic biopolymer obtained from renewable resources—and due its biodegradability, is also widely used in medicine. In this study, films and multifilament yarns from modified biodegradable PLA granules with ethanolic and aqueous myrrh extracts were developed and characterized. Optical microscopy was used to determine the surface morphology of PLA/myrrh multifilament yarns. Tensile tests, ultraviolet-visible (UV-vis), differential scanning calorimetry (DSC) were applied to determine, consequently, mechanical, optical properties and degree of crystallinity of PLA/myrrh films and multifilament yarns. The chemical composition of PLA/myrrh multifilament yarns was estimated by Fourier-transform infrared (FTIR) spectroscopy method. The results showed that it is possible to form PLA melt-spun multifilament yarns with myrrh extract. The type of myrrh extract (ethanolic or aqueous) has a significant influence on the mechanical and optical properties of the PLA films and melt-spun yarns. The mechanical properties of PLA films and melt-spun multifilament yarns formed from PLA granules with aqueous myrrh extract decreased 19% and 21% more than PLA with ethanolic extract, respectively. Analysis of UV-vis spectra showed that, due to the yellow hue, the reflectance of PLA films and melt-spun PLA multifilament yarns modified with myrrh extracts decreased exponentially. The DSC test showed that multifilament yarns from PLA modified with aqueous extract had the highest degree of crystallization.

## 1. Introduction

Melt spinning or hot melt extrusion, is one of the most widely used processes to produce polymeric filaments. It is an extremely promising technique and includes advantages such as continuous processing, easy scalability, solvent-free fabrication and fairly low production costs [1,2,3,4,5]. The primary process variables of melt spinning are extrusion temperature; mass throughput per spinneret hole; cooling conditions; size and shape of the spinneret holes; spin line length and take-up velocity of the filaments or filament drawing ratio. These process variables influence the structure and properties of the melt-spun filaments [6].

Polylactide (PLA) is a thermoplastic synthetic biopolymer with unique bio-properties including biocompatibility, biodegradability and antibacterial activity, due to which it is widely used for biomedical application (such as musculoskeletal injuries, tissue engineering, drug release systems) [7,8,9]. PLA melt-spun yarns are widely investigated in order to improve and develop new functional properties for biocompatible and biodegradable yarns. M. Naeimirada et al. [5] investigated the production of melt-spun hollow PLA fibers. They estimated that throughput of PLA polymer and winding speed of fibers have a significant influence on fiber cross section, linear density and tensile properties, though the extrusion temperature and quench flow caused marginal changes to the internal diameter, tensile strength and tenacity of fibers. Such PLA hollow fibers may be used for drug transportation in medicine and agriculture. Y. Kawahara and coauthors [7] investigated bicomponent PLA/poly (butylene terephthalate) (PBT) melt-spun fibers for their application in medical clothing materials. Bicomponent fibers (core: PBT, skin: PLA) have lower tensile properties than pure PLA or PBT fibers. M. Maqsood et al. [8] studied manufactured PLA multifilament yarns containing phosphorous–nitrogen-based flame retardant (EXP) as an acidic source, together with kraft lignin (KL) as carbonic source. It was estimated that with higher content of KL and EXP, elongation at the break of multifilament yarns was decreased and yarns with lower mechanical properties were obtained [8]. The same results were noted when analyzing PLA with soy fillers melt-spun fibers which may be used for biodegradable nonwovens. It was estimated that the addition of soy fillers to PLA slightly decreased the tensile properties of fibers, though tensile modules do not change [10]. A. Mujica-Garcia and coauthors [11] estimated that the tensile properties and thermal stability of melt-spun PLA/cellulose nanocrystals (PLA/CNC) fibers (with a 1% weight fraction of nanocrystals) would significantly improve with the addition of CNC. A.S. Doumbia et al. [12] investigated the influence of ZnO on the structure of antibacterial PLA melt-spun yarns. They analyzed two types of ZnO nanoparticle: those with surface treatment (ZnO_T_) and those without surface treatment (ZnO_NT_), with concentration of 1% and 3% nanoparticles. PLA multifilament yarns with 3% of ZnO_NT_ exhibited the lowest mechanical properties.

Natural compounds from plants demonstrate antibacterial, antifungal and antioxidant activity. PLA films with incorporated bergamot, lemongrass, rosemary, clove, oregano, thymol essential oils and cinnamon oil, as well as grapefruit seed extract, were developed for their application in active packaging materials [13,14,15,16,17]. The beneficial properties of natural products can be introduced to textiles during their manufacturing process or during finishing. This resulted in the successful formation of polypropylene (PP) melt-spun multifilament yarns with propolis extract [18] or PET braided yarns with *Laurus nobilis* [19]. M. Kanerva and coauthors [20] formed polyethylene (PE), PP, PLA, polyamide (PA) and cornstarch-based biopolymer (CS) melt-spun yarns with pine rosin. For all fibers, the addition of rosin tended to decrease the initial stiffness of the yarns. It was estimated that the antibacterial properties of melt-spun yarns against *S. aureus* and against *E. coli* were dependent on the selected polymer type.

Another beneficial natural material is Myrrh, which has long been used as a medicine and wound dressing. It has good antimicrobial effects against *Staphylococcus aureus*, *Salmonella enterica* serovar, *Typhimurium* strains, *Klebsiella pneumoniae*, *Serratia marcescens*, *Enterobacter cloacae*, *Pseudomonas aeruginosa*, *Providencia stuarti, Escherichia coli*. Myrrh is an aromatic gum resin obtained from a small tree found in northeast tropical Africa, Arabia and the Indian subcontinent. Compositionally, myrrh consists of alcohol-soluble resins (25–40%), volatile oils (3–8%) and a water-soluble gum (30–60%) [21,22,23,24]. It was estimated that myrrh extract produces good color fastness properties on wool and silk fabrics and improves the tensile strength of analyzed samples [21]. According H.A. Al Alamoudi and A.A. Salem [21] research of natural products use in textiles are very pure poor, although natural products may significantly improve the functional properties of textiles. Due these reasons, the modification of textile raw materials or final textile products with natural materials must be investigated.

The aim of this research is to investigate the possibilities of forming biodegradable melt-spun multifilament PLA yarns with myrrh resin. In order to investigate the influence of myrrh extract on the PLA melt spinning process, two types of myrrh extracts (ethanolic and aqueous) were analyzed. The influence of myrrh extracts on PLA melt-spun yarns and PLA films structure, mechanical and thermal properties were investigated in this research.

## 2. Materials and Methods

### 2.1. Materials

Films and multifilament yarns were formed from the polylactic acid (PLA) 6100D (Nature Works, Blair, NE, USA). Its glass transition temperature is 55–60 °C and melting temperature is 165–180 °C [25]. Myrrh resin was imported from India (Ekokolekcija, Vilnius, Lithuania). Ethanol (96%) for extraction was purchased from Stumbras (Kaunas, Lithuania).

### 2.2. Preparation of Myrrh Ethanolic Extract

Solid particles of myrrh rosin were crushed to a fine powder before the extraction process. For extraction of raw myrrh, 96% ethanol was used. Mass ratio of myrrh rosin and ethanol in extraction mixture was 30/70. Myrrh ethanolic extract was produced for 12 h in a round bottom flask at boiling point of ethanol (approximately at 78 °C). Gentle boiling of ethanolic extraction mixture was ensured by heating at 90 °C in the sand bath. The mixture was stirred with a magnetic stirrer at 400 rpm (IKA RH, basic KT/C, Staufen, Germany) during whole extraction period. The reflux condenser (Allihn type, Schott AG, Mainz, Germany) was used to prevent solvent evaporation from the boiled ethanolic myrrh extract. Myrrh ethanolic extract was filtered by Filtrak No. 389 filter paper using a Buchner funnel to remove the undissolved solid particles (sand and ground myrrh).

### 2.3. Preparation of Myrrh Aqueous Extract

Solid particles of myrrh rosin were crushed into a fine powder before the extraction process. For the extraction of raw myrrh distilled water was used. Mass ratio of myrrh rosin and distilled water in extraction mixture was 10/90. Myrrh aqueous extract was produced at 95 °C for 90 min, in round bottom flask with ball refrigerator (Allihn), in sand bath, while stirring with a magnetic stirrer (IKA RH, basic KT/C, Staufen, Germany). The stirrer rotation speed was 400 rpm. Myrrh aqueous extract was centrifuged at 2500 rpm for 5 min and then filtered by Filtrak No. 389 filter paper using a Buchner funnel to remove the undissolved solid particles.

### 2.4. Modification of PLA Granules with Myrrh Extracts

The PLA granules were modified with myrrh extract (ethanolic and aqueous) by the spraying process. PLA granules were covered with myrrh extracts, mixed and dried at a temperature of 80 °C for at least 60 min until the solvent (ethanol or water) had evaporated. Such procedure was repeated four time, while bicomponent PLA/Myrrh extract granules of 99.5/0.5 wt/wt were formed.

### 2.5. Melt Spinning of PLA Multifilament Yarns

**Figure 1:**
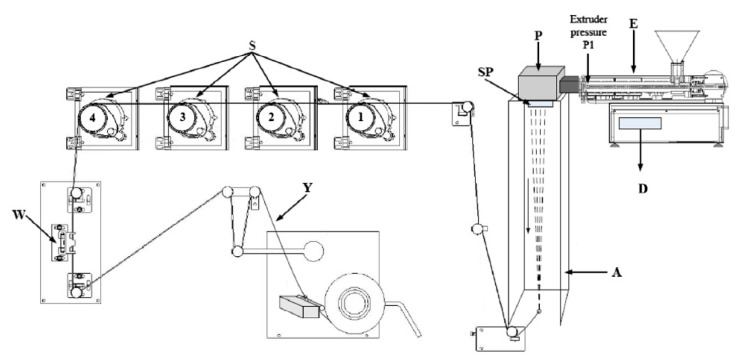
Principal scheme of the laboratory spinning equipment COLLIN^®^ CMF 100 (Dr. Collin GmbH, Maitenbeth, Germany): E—extruder; P—melting pump; SP—spinneret; A—air quench cabinet; D—display; S—stretching gadgets; W—whirling unit; Y—multifilament yarn from microfibers [26].

Multifilament yarns from PLA polymer (A yarns), PLA modified with myrrh ethanolic extract (B yarns) and aqueous extract (C yarns) were manufactured by COLLIN^®^ CMF 100 (Dr. Collin GmbH, Maitenbeth, Germany) single-screw extruder equipment. The single-screw extruder (L/D = 25:1) has seven heating zones, where the temperature during experiments was set to 188 °C. The average speed of extruder was set to 29 rpm. The circular spinnerets (Figure 1, indicated by SP) with 24 holes (diameter 0.45 mm) were used during these experiments. Cooling of filaments (Figure 1, indicated by A) was achieved with cross-flow air quenching at the temperature of 14 °C. The temperature of the stretching rolls was as follows: S1 = 69 °C; S2 = 75 °C; S3 = 78 °C and S4 = 85 °C. The speed of stretching rolls was as follows S1 = 99 rpm; S2 = 198 rpm; S3 = 228 rpm and S4 = 250 rpm (Figure 1), with a drawing ratio of 2.53.

### 2.6. Formation of PLA Films

Films from pure PLA (A film), PLA modified with myrrh ethanolic extract (B film) and aqueous extract (C film) were formed using laboratory hydraulic press the Joos Laboratory Press LAP40 (Gottfried Joos GmbH & Co. KG, Pfalzgrafenweiler, Germany), at 2.83 MPa pressure, at a temperature of 180 °C. The thickness of the films formed was: A—0.17 ± 0.01 mm; B—0.41 ± 0.02 mm; C—0.35 ± 0.04 mm.

### 2.7. Linear Density of Yarns

Specimens of 50 m in length were prepared by reeling skeins with Zweigle L232 (Zweigle Textilprüfmaschinen GmbH & Co. KG, Reutlingen, Germany), for the estimation of the linear density of multifilament yarns. The mass of skeins was determined under the standard atmospheric conditions. The linear density of multifilament yarns was calculated according to Equation (1):

(1) T = m/l

tex where *m*—mass, g and *l*—length, km of 50 m specimen.

The test result was calculated as average of five measurements.

### 2.8. Structure of PLA Multifilament Yarns

The structure of PLA multifilament yarns was investigated by optical microscope Olympus SZX7 (Olympus Corp., Tokyo, Japan), at a magnification ×56. The structure of melt-spun PLA multifilament yarn was investigated in at least in five different randomly chosen places.

### 2.9. Mechanical Properties of PLA Films and Multifilament Yarns

The mechanical properties (breaking stress and elongation at break) of the prepared films were determined according to EN ISO 527–1:2012 standard. Using a universal testing machine Tinius Olsen H10KT (Tinius Olsen, Inc., Salford, UK) equipped with a 500-N load cell. The specimens were cut into rectangular strips 150 × 10-mm size. The length between clamps was 100 mm, and the stretching speed was 10 mm/min. The number of tensile tests was 5 for each sample.

Mechanical properties (the breaking force and elongation at break) of PLA multifilament yarns were determined according to the EN ISO 2062:2009 standard. Experiments were carried out in a standard atmosphere where the temperature was 20 ± 2 °C and relative humidity was 65 ± 4%. A universal testing equipment Zwick/Z005 (Zwick GmbH & Co. KG, Ulm, Germany) with operating program testXpert^®^ was used. The length between clamps was 100 mm and the stretching speed was 500 mm/min. The number of tensile tests from a package was 35.

### 2.10. Optical Properties of PLA Films and Multifilament Yarns

The UV-vis diffuse reflectance spectra of PLA films (A, B and C films) and melt-spun multifilament yarns (A, B and C yarns) were measured with a Lambda 35 UV-vis spectrometer (PerkinElmer, Waltham, MA, USA) at a wavelength range of 200–800 nm. The thickness of PLA films analyzed was approximately 0.5 mm. Diffuse reflectance measurements of PLA multifilament yarns were performed by wrapping multiple layers (no less than 10) around a glass coverslip used in microscopy.

### 2.11. Thermal Behavior of PLA Films and Multifilament Yarns

Differential scanning calorimetry (DSC) analysis using a Netzsch Polyma DSC 214 (NETZSCH-Gerätebau GmbH, Selb, Germany) was conducted for PLA films (A, B and C films) and PLA multifilament yarns (A, B and C yarns) to obtain the glass transition, melting, crystallization and cold crystallization temperatures of the specimens. Aluminum pans were used for each sample with an average sample mass of 5 mg. The heating and cooling scan rates were 10 °C/min, under a nitrogen atmosphere at a flow rate of 20 mL/min. Test specimens were heated and cooled during DSC analyses, within a certain temperature interval ranging from 15 °C to 240 °C and back to 15 °C. The degree of crystallinity of pure PLA and PLA saturated with myrrh samples was calculated using the following Equation (2) [27].

(2) $$x_{c}\left(\%\right)\;=\;\frac{\Delta Hm\;-\Delta Hc}{\lambda \Delta Hm,o}\times 100\%$$

where Δ*H*_m_—melting enthalpy of PLA (J/g); Δ*H*_c_—cold crystallization enthalpy of PLA (J/g); *λ*—mass fraction of PLA in composite yarns; Δ*H*_m,o_—melting enthalpy of 100% crystal, the value was 93.6 J/g [28].

### 2.12. Chemical Interactions between PLA and Myrrh Extracts

Fourier-transform infrared spectroscopy (FTIR) using a VERTEX 70 (Bruker Co., Ettlingen, Germany) was used in order to determine possible interactions between functional groups of myrrh extracts and PLA. PLA multifilament yarns (A, B and C yarns) were investigated using the devices of attenuated total reflectance (ATR) mode, equipped with a diamond crystal (Bruker Co., Ettlingen, Germany). The measured wavenumber range was 400–4000 cm^−1^, with a resolution of 2 cm^−1^.

### 2.13. Statistical Analysis

In order to investigate the influence of the modification of PLA granules by myrrh extracts on the linear density melt-spun multifilament yarns and mechanical properties of films and melt-spun multifilament yarns, difference between data average was estimated by Student’s *t*-test.

## 3. Results and Discussion

### 3.1. The Influence of Myrrh Extract on the Structure and Mechanical Properties of Melt-Spun PLA Multifilament Yarns

**Figure 2:**
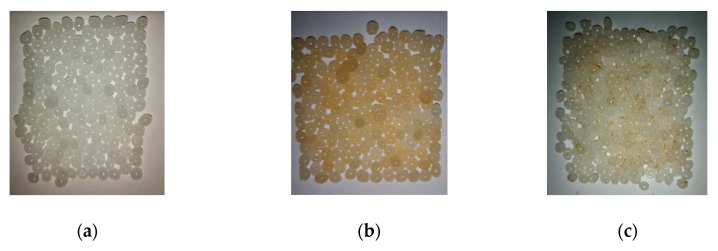
Images of (**a**) pure polylactide (PLA) granules; (**b**) PLA granules modified with myrrh ethanolic extract; (**c**) PLA granules modified with myrrh aqueous extract.

The main challenge in the modification of hydrophobic PLA polymeric granules was related to the formation of a smooth cover of myrrh components on granules. It was estimated that it was possible to form smooth cover (film) on PLA granules using myrrh ethanolic extract and PLA granules with myrrh powder using myrrh aqueous extract (Figure 2).

**Figure 3:**
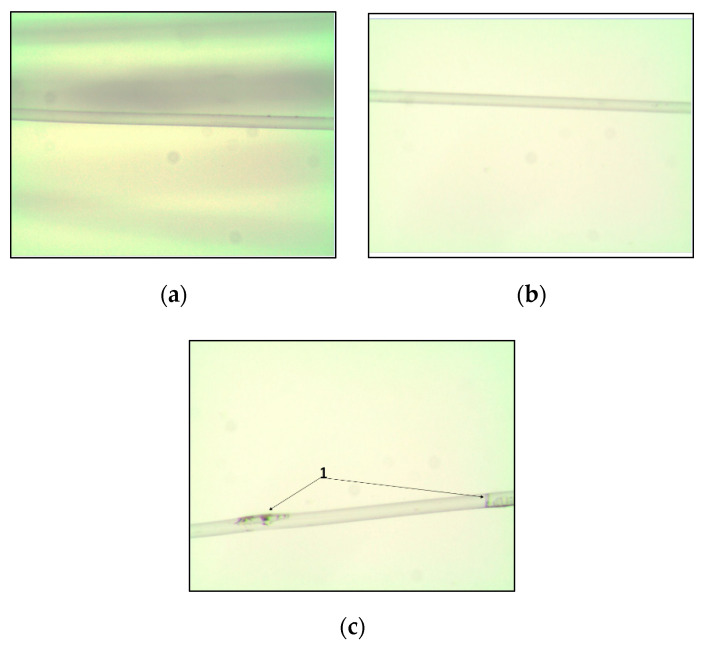
Images of one filament from melt-spun PLA multifilament yarns formed from (**a**) pure PLA polymer (A yarns), (**b**) PLA polymer modified with myrrh ethanolic extract (B yarns), (**c**) PLA polymer modified with myrrh aqueous extract (C yarn). Number 1 indicates unmelted myrrh aqueous extract derivative.

In Figure 3, images of one filament (from 24 filaments) of pure melt-spun PLA multifilament yarns (a) A yarn, PLA yarns modified with ethanolic extract (b) B yarn and PLA yarns with aqueous extract (c) C yarn are presented. From these images, it is possible to observe that C yarns consist of unmelted myrrh resin derivatives (Figure 3, indicated with number 1), which were not found in yarns produced from PLA polymer modified with ethanolic extract.

The linear density (tex) of melt-spun yarns depends on their raw material and technological parameters (melting temperature, pressure, drawing and winding) [6,27,29]. From the results presented in Figure 3a, it is possible to notice that the linear density of melt-spun yarns from PLA modified with aqueous myrrh extract (C) decrease by approximately 26%, while linear density of yarns from PLA with ethanolic myrrh extract varied in their margins of error. Whereas the technological parameters of melt spinning were the same for all samples, it is possible to make the assumption that the modification of PLA granules with aqueous myrrh extract changed the properties of the melt. In order to prove such assumption, the melt flow (*α*, g/10 min) of pure PLA and modified PLA was estimated by extruder plastomer (AB Lituanica, Kaunas, Lithuania). Melted (at 180 °C) polymer had an outflow through calibrated capillary at 22-N load and it was estimated that melt flow of pure PLA—41 ± 4 g/10 min; PLA modified with ethanolic myrrh extract—41 ± 2 g/10 min and PLA modified with aqueous myrrh extract—34 ± 1 g/10 min. The modification of PLA with aqueous myrrh extract decreased the PLA melt flow by approximately 17%, due to which the linear density of melt-spun multifilament yarns also decreased. Comparing the data of linear density according to the Student’s *t*-test, it was estimated that the difference between linear density of A and B yarns is not significant *t*_T A–B, tex_ = 1.63, but between A and C yarns it is meaningful *t*_T A–C, tex_ = 31.

**Figure 4:**
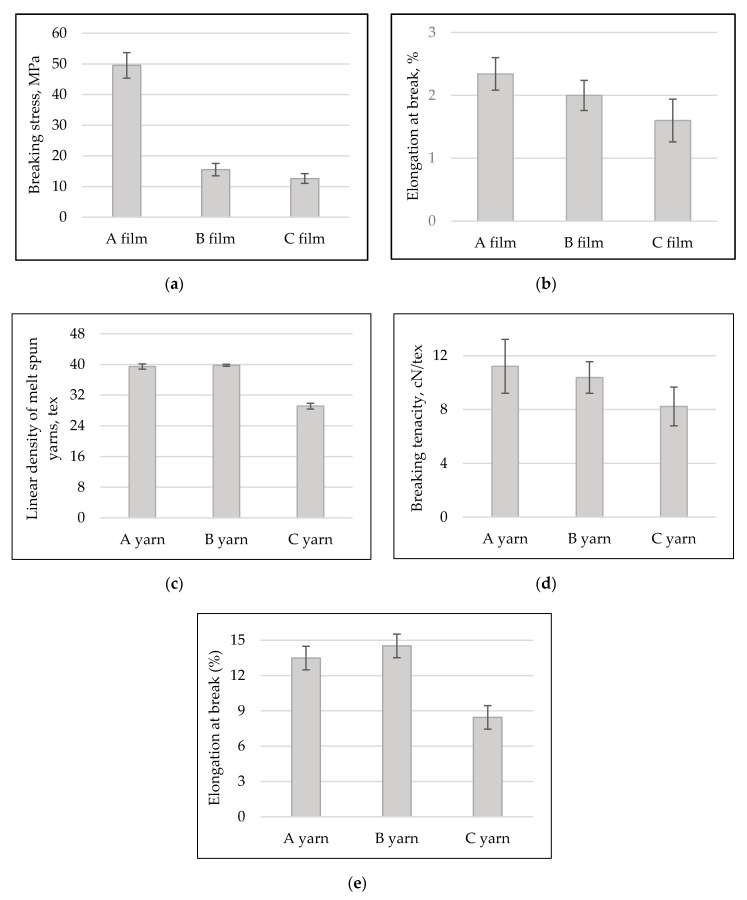
Influence of the modification of PLA granules with myrrh extract on: (**a**) PLA films breaking stress, MPa; (**b**) elongation at break, %; (**c**) melt-spun multifilament yarns linear density, tex; (**d**) breaking tenacity, cN/tex and (**e**) elongation at break, %.

Tensile tests on PLA films and melt-spun PLA yarns were estimated to determine how the mechanical properties of films and yarns were influenced by the presence of myrrh extract. Comparing the mechanical properties of PLA films (Figure 4a,b), it was observed that the films with extracts had inferior properties. The breaking stress (*σ*, MPa) of the B and C films decreased by 70% and 75%, elongation at break (*ε*_f_,%)—21% and 30%, respectively. Estimated Student’s *t*-test values *tσ*_A-B, MPa_ = 5.6; *t_ε_*_A-B, %_ = 6.5, *tσ*_A-C, MPa_ = 4.3; *t_ε_*_A-C,%_ = 19.9 show that modification of PLA granules with myrrh extract have significant influence on mechanical properties of melted PLA films. It was estimated that inserted oregano essential and cinnamon oil materials act as a plasticizers in PLA films to increase PLA chain mobility and reducing the mechanical properties of films [16,17]. It is possible to do assumption that due the same reason and myrrh reduce the mechanical properties of PLA films.

The tensile properties of melt-spun fibers (Figure 4d,e) depend on the internal structure of the fiber (strongly depends on linear density of multifilament yarns (diameter of fibers)) and are sensitive to spinning conditions [18,30]. When the linear density of yarn C decreased, consequently, the breaking tenacity (*f*_y_, cN/tex) of melt-spun yarns decreased by approximately 26% and elongation at break (*ε*_y_, %) also decreased 37%. While the breaking tenacity (*f*_y_, cN/tex) of B yarns decreased by only 7%, elongation at break (*ε*_y_, %) increased by 8%. Comparing the results of the breaking tenacity (*f*_y_, cN/tex) and elongation at break (*ε*_y_, %) of yarns A and B according to the Students *t*-test was resulted in value of *t*_f A_-_B, cN/tex_ = 1.19; *t_ε_*_A-B, %_ = 2.17 and for yarns A and C: *t*_f A-C, cN/tex_ = 3.9; *t_ε_*_A-C, %_ = 11.4. The modification of PLA granules with myrrh extract influenced the mechanical properties of melt-spun yarns. In this study, a smooth film was formed on PLA granules using myrrh ethanolic extract (B) and on the PLA granules with myrrh powder (C) using myrrh aqueous extract (see Figure 2). It is known that the insertion of fillers (nanoparticles, crushed particles) in melt-spun yarns decreases their mechanical properties [10,12,20]. Due to the huge number of unmelted myrrh particles, melt-spun yarns with aqueous myrrh extract (C yarns) exhibit significantly lower mechanical properties and fragility, and such yarns would therefore not be suitable for use in further processes (knitting, weaving, braiding, etc.).

### 3.2. Optical Analysis of PLA Films and Melt-Spun Multifilament Yarns

**Figure 5:**
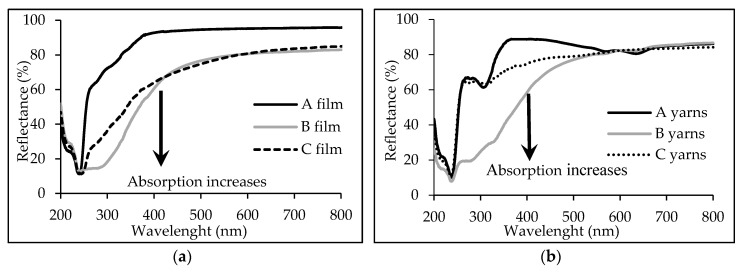
UV-vis spectra analysis of: (**a**) PLA films (A—pure PLA polymer; B—PLA polymer modified with myrrh ethanolic extract; C—PLA polymer modified with myrrh aqueous extract) and (**b**) PLA multifilament yarn from (A—pure PLA polymer; B—PLA polymer modified with ethanolic myrrh extract; C—PLA polymer modified with aqueous myrrh extract).

UV-vis spectra of semi-opaque films made from pure PLA (A film) uniformly demonstrate and highest diffuse reflectance (lowest absorbance) in the whole visible and near-UV (up 380 nm) spectral region. The reflectance of this film decreases evenly in the range of wavelength from 380 nm to 260 nm and reaches its minimum peak at 245 nm (Figure 5a). This peak is caused by the strong absorption of UV radiation by ester groups of polylactide chains, and it is observed in all spectra.

Meanwhile, the films made from modified PLA with myrrh extracts (B and C films) are opaque and also have almost uniform reflectance, but approximately 15% lower, in spectral range from 800 nm to 550 nm. With the decreasing wavelength in the rest of visible spectrum, the reflectance of modified PLA films (B and C films) decreases exponentially, and this is related to their yellow hue. In the UV region (from 400 nm to 250 nm) of spectra of modified PLA films (B and C films) broad absorption (decreased reflection) bands are observed. All these spectral differences, in comparison with pure PLA film (A film), are most likely due to the organic additives of the myrrh extract in the modified PLA films (B and C films). It should be noted that the most significant decrease of diffuse reflectance (highest absorbance) is characteristic of a film made from PLA modified with ethanolic myrrh extract (B film), and this could be related to higher concentrations of extracted organic compounds in such film.

UV-vis spectral analysis of melt-spun PLA multifilament yarns made from pure (A yarn) and modified PLA (B and C yarns) showed some similar features to PLA films. In all spectra of melt-spun A multifilament yarns are observed the same and uniform, but in comparison with pure PLA films (A films), slightly lower diffuse reflectance in the range of wavelength from 800 nm to 550 nm in evident. Most probably, the decreasing of diffuse reflectance is due to surface roughness of the wrapped layer of PLA multifilament yarns analyzed. In the spectra of pure PLA multifilament yarns, the second absorbance peak (decreasing of reflectance) is clearly identified at a wavelength of 310 nm. This peak could be attributed to ethylene copolymer additive in PLA, which usually is added at the 1–5% level to improve the toughness, flexibility and impact strength of PLA parts produced by the thermoforming and injection molding techniques [31]. The presence of compounds introduced into PLA multifilament yarns by the addition of ethanolic myrrh extract (B yarns) also indicate the exponential increase of absorption (decreasing of diffuse reflection) in the range of wavelength from 550 nm to 400 nm, as well as broad and intensive absorption band in the entire UV region of recorded spectrum.

### 3.3. Thermal Behavior of PLA Films and PLA Melt-Spun Multifilament Yarns

**Figure 6:**
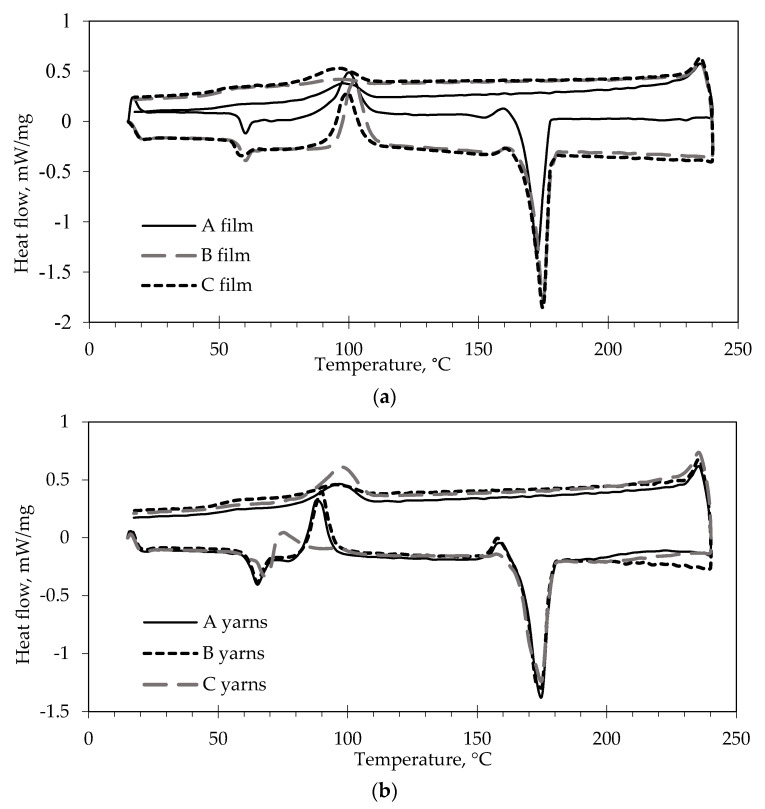
DSC thermograms analysis of (**a**) PLA films (A—pure PLA polymer; B—PLA polymer modified with ethanolic myrrh extract; C—PLA polymer modified with aqueous myrrh extract) and (**b**) PLA multifilament yarn (from A—pure PLA polymer; B—PLA polymer modified with ethanolic myrrh extract; C—PLA polymer modified with aqueous myrrh extract).

DSC testing was applied to analyze the thermal properties and crystallinity of the PLA films and yarns produced. According to the thermograms presented in Figure 6 and data in Table 1, it is possible to notice that the PLA film sample (A film) and PLA containing myrrh extracts (B and C films) demonstrated similar thermal behavior. The glass transition temperature *T*_g_ (58.7–59.8 °C) and melting-point temperature *T*_m_ (173–174.9 °C) are similar in almost all films samples, only film B (PLA modified with ethanolic myrrh extract) shows a low degree of crystallinity *X*_c (B film)_ = 18.1%. Analyzing the thermograms of melt-spun multifilament yarns, it is possible to observe that multifilament yarns from PLA modified with aqueous myrrh extract (C yarns) do not have high crystallization temperature (*T*_c2_) and their first crystallization degree *T*_c1(C yarns)_ = 74.5 °C is around 15 °C lower than of A and B yarns (*T*_c1(A yarns)_ = 88.5 °C, *T*_c1(B yarns)_ = 89.6 °C). C yarns have the highest degree of crystallization *X*_c (C yarn)_ = 44.3%. The decrease in the *T_c1_* can be attributed to greater molecular chain mobility in PLA modified with aqueous myrrh extract. M. Maqsood [32] estimated that the increase in the *T_c_* of melt-spun PLA fibers due to higher concentration of oxidized starch can be correlated with quite high agglomeration of the additives which decreases the molecular chain mobility. Analyzing plasticized PLA films with different epoxidized karanja oil content [33] was concluded that *T_c_* decrease due to greater mobility of the polymer chains due to the oil plasticizing effect. Either according K.M. Zakir Hossain et al. study [34] a higher degree of chain orientation in PLA polymer have fibers with smaller diameters. Melt spun PLA filaments with the lower diameter have higher degree of crystallization. It was proved that the increase of degree of crystallinity is due to the increase of the draw ratio of PLA filaments (the higher the draw ratio, the lower the diameter of filaments) which implies an improvement of the molecular orientation. The molecular orientation is known to promote crystallization [35]. Multifilament yarns from PLA modified with aqueous myrrh extract (C yarns) have the smallest linear density (tex) (i.e., the thinnest filaments), consequently, exhibit higher degree of molecular orientation and have the highest degree of crystallization. In the research X. Yuan and coauthors [9], two crystallization peaks were also not noted in the thermograms of hot-drawn PLA fibers due to high crystallinity. Comparing the data from the thermograms of films and yarns, it was estimated that melt-spun PLA yarns have higher glass transition temperature *T*_g_, a lower first crystallization temperature *T*_c1_ and a higher degree of crystallization *X*_c_ than films, but the same melting temperature. It is possible to explain such results based on the improvement of the molecular orientation of the PLA polymer under intense stretching during the formation of multifilament yarns. The results obtained—that the drawing process enhanced the structural properties of melt-spun—correlate with others researchers results [18,29].

When analyzing DSC cooling curves (Figure 6a,b), it is possible to notice that myrrh extracts do not significantly influence the peak crystallization temperature on cooling (*T*_cc_) for films nor for yarns. These results are similar to another study [36], were the *T*_cc_ of pure PLA was estimated at 93.8 °C.

### 3.4. FTIR Analysis

**Figure 7:**
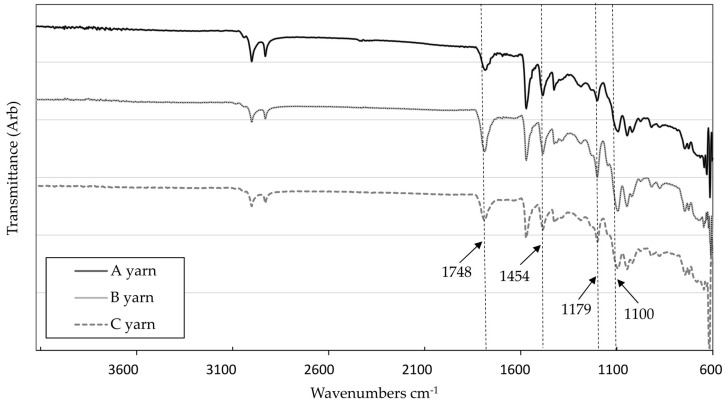
ATR–FTIR spectrum of different PLA multifilament samples. A—pure PLA polymer; B—PLA polymer modified with ethanolic myrrh extract; C—PLA polymer modified with aqueous myrrh extract.

The chemical structure of samples was investigated with FTIR. Figure 7 shows the FTIR spectra of PLA yarns samples A–C. The shifts and typical peaks of pure PLA fibers were compared with available literature [28,37,38]. The main characteristics of PLA peaks were observed at three prominent—regions and were found to be comparable to several FTIR studies. The peak observed at 1748–1751 cm^−1^ is assigned as carbonyl stretching C=O in the CO–O groups of PLA. The second needle-like peak found in the region 1179–1180 cm^−1^ is the stretching vibration absorption peak of “–C–O–” bond in ester groups in PLA chains. The region of third peak found consistently in all FTIR results (1127–1043 cm^−1^) represents the stretching vibration of C–O in CH–O groups in PLA chains. The deformation vibration absorption peak of–CH_3_ groups at 1454 cm^−1^ [28,37].

The shift of the bands was not identified when the possible interaction of differently modified PLA was analyzed. The comparison between the ATR–FTIR spectra of modified PLA multifilament and those of pure PLA revealed no significant differences that could indicate the presence of impact between PLA and different myrrh extracts in multifilament produced.

## 4. Conclusions

The results of the study have confirmed the possibility of incorporating myrrh extracts into PLA multifilament yarns and films. The modification of PLA granules with myrrh extracts (aqueous and ethanolic) caused an decrease in breaking stress (MPa) and elongation at break (%) of PLA films. Due to the lowest melt flow index (g/10 min) and presence of unmelted myrrh resin derivatives, melt-spun multifilament yarns formed from PLA with aqueous myrrh extract have lower linear density (tex), breaking tenacity (cN/tex) and elongation at break (%) nor yarns formed from pure PLA and PLA modified with ethanolic myrrh extract. The film and multifilament yarns made from PLA modified with ethanolic myrrh extract exhibit the most significant decrease in diffuse reflectance, and it could be related to higher concentrations of extracted organic compounds in such materials. The drawing process of melt-spun multifilament yarns improves their molecular orientation and due to this process melt-spun yarns have higher degree of crystallinity nor films from the same polymer type. FTIR spectra revealed no significant differences that could indicate the presence of impact between PLA and different myrrh extract in the multifilament produced. Summarizing the results of the formation process and the mechanical, thermal and optical properties of yarn produced from PLA with myrrh extract, it possible to conclude that, for formation of melt-spun yarns, ethanolic myrrh extract should be used and after additional investigation (antimicrobial, kinetic release of myrrh’s biologic active compounds) such multifilament yarns may be used for biomedical applications.

## Figures and Tables

**Table 1:** Differential scanning calorimetry (DSC) analysis of pure PLA and modified PLA with myrrh extracts films and multifilament yarns.

Code of Sample	*T*_g_ (°C)	*T*_c1_ (°C)	*T*_c2_ (°C)	∆*H*_c_ (J/g)	*T*_cc_ (°C)	*T*_m_ (°C)	Crystallinity *X*_c_ (wt%)
A film	59.8	100.0	159.6	27.1	97.7	173	28.1
B film	59.7	101.7	161.7	34.2	96	174.9	18.1
C film	58.7	99.2	160.7	30.3	96.3	174.5	27.9
A yarn	65.6	88.5	158.8	23.9	96.9	174.5	39.5
B yarn	65.3	89.6	158.1	25.0	95.3	174.0	40.4
C yarn	68.6	74.5	–	16.7	97.7	174.9	44.3
